# Application of the U-Net Deep Learning Model for Segmenting Single-Photon Emission Computed Tomography Myocardial Perfusion Images

**DOI:** 10.3390/diagnostics14242865

**Published:** 2024-12-20

**Authors:** Ahmad Alenezi, Ali Mayya, Mahdi Alajmi, Wegdan Almutairi, Dana Alaradah, Hamad Alhamad

**Affiliations:** 1 Radiologic Sciences Department, Kuwait University, Kuwait City 31470, Kuwait; 2 Computers and Automatic Control Engineering Department, Tishreen University, Latakia 2230, Syria; 3 Nuclear Medicine Department, Ministry of Health, Jahra Hospital, Al Jahra 03200, Kuwait; abndlmh89@gmail.com; 4 Faculty of Allied Health, Kuwait University, Kuwait City 31470, Kuwait; wgdan.almutairi@hsca.ku.edu.kw (W.A.); danah.alaradah@hsca.ku.edu.kw (D.A.); 5 Occupational Therapy Department, Kuwait University, Jabriya 31470, Kuwait; h.alhamad@ku.edu.kw

**Keywords:** artificial intelligence, deep learning, image segmentation, SPECT, MPI U-Net

## Abstract

Background: Myocardial perfusion imaging (MPI) is a type of single-photon emission computed tomography (SPECT) used to evaluate patients with suspected or confirmed coronary artery disease (CAD). Detection and diagnosis of CAD are complex processes requiring precise and accurate image processing. Proper segmentation is critical for accurate diagnosis, but segmentation issues can pose significant challenges, leading to diagnostic difficulties. Machine learning (ML) algorithms have demonstrated superior performance in addressing segmentation problems. Methods: In this study, a deep learning (DL) algorithm, U-Net, was employed to enhance segmentation accuracy for image segmentation in MPI. Data were collected from 1100 patients who underwent MPI studies at Al-Jahra Hospital between 2015 and 2024. To train the U-Net model, 100 studies were segmented by nuclear medicine (NM) experts to create a ground truth (gold-standard coordinates). The dataset was divided into a training set (*n* = 100 images) and a validation set (*n* = 900 images). The performance of the U-Net model was evaluated using multiple cross-validation metrics, including accuracy, precision, intersection over union (IOU), recall, and F1 score. Result: A dataset of 4560 images and corresponding masks was generated. Both holdout and k-fold (k = 5) validation strategies were applied, utilizing cross-entropy and Dice score as evaluation metrics. The best results were achieved with the holdout split and cross-entropy loss function, yielding a test accuracy of 98.9%, a test IOU of 89.6%, and a test Dice coefficient of 94%. The k-fold validation scenario provided a more balanced true positive and false positive rate. The U-Net segmentation results were comparable to those produced by expert nuclear medicine technologists, with no significant difference (*p* = 0.1). Conclusions: The findings demonstrate that the U-Net model effectively addresses some segmentation challenges in MPI, facilitating improved diagnosis and analysis of mega data.

## 1. Introduction

Cardiovascular diseases (CVDs) continue to pose a significant global health challenge, representing the leading cause of mortality worldwide and resulting in a staggering number of deaths each year. The World Health Organization (WHO) reports that CVD accounts for more than 70% of Kuwaiti population mortality [1]. Thus, accurate imaging and diagnosis are crucial to a successful treatment plan. Myocardial perfusion imaging (MPI) is a crucial nuclear medicine procedure in cardiovascular imaging, playing an important role in diagnosing and assessing the risk of patients with coronary artery disease (CAD) [2,3,4,5,6]. Despite its good diagnostic utility, accurate interpretation is still challenging [4] due to problems related to image analysis and segmentation owing to limited image quality (e.g., limited resolution and inherent noise).

Additionally, some apparent attenuation defects (e.g., in the inferior wall caused by diaphragmatic motion) can cause false positive results [5,6]. Moreover, errors and inter-observer variability in processing may occur in the absence of unified automated segmentation and might be exaggerated during high workloads in nuclear medicine departments [5,7]. However, these problems and segmentation errors can be avoided and imitated by utilizing artificial intelligence (AI) algorithms [8,9].

In recent years, AI has enhanced the consistency and the outcome segmentation process via computation and mathematical models that mimic human intelligence [10,11,12]. Machine learning (ML) is a subset of AI models that include a variety of approaches to accomplish specific tasks using data (e.g., segmentation) [8,11]. It has acquired attention in nuclear medicine imaging, shifting from conventional data analysis to more advanced image analysis in diagnostic imaging and MPI [10,11]. By training machine learning (ML) on extensive datasets to identify intricate patterns and connections in medical images, this can facilitate automated image segmentation. This automatic segmentation might overcome the inconsistency problem and provide more accurate segmentation. To reach this outcome, the U-Net model, a remarkable ML architecture, has become widely recognized in image segmentation tasks and disease classification [10,11]. This architecture is characterized by its simplicity of use and high accuracy in image segmentation.

**Figure 1:**
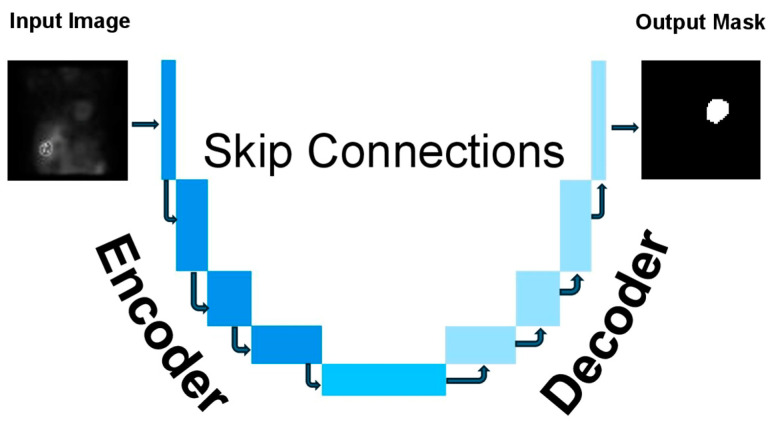
The structure of the U-net model represents the input image (original image) and output mask.

Additionally, its encoder–decoder and skip connection structures allow it to accurately record specific details from input images to learn and implement learning in new datasets [13]. The model’s U-shape construction gives rise to its name (see Figure 1), which describes the contracting paths (encoders) followed by expansion paths (decoders) of the learning nodes within its architecture [13]. The encoder extracts features from the input image and reduces its resolution, while the decoder restores the image to its original size and generates the segmentation map. By integrating these two paths, U-Net effectively performs high-accuracy segmentation tasks facilitated by skip connections [14,15]. Additionally, U-Net preserves spatial information lost during segmentation, guaranteeing that the segmentation process keeps the complex anatomical features of the source images. This feature improves the consistency of the segmentation results, boosting their clinical value [13,14,15,16].

This study aims to use U-Net machine learning for standardized and accurate image segmentation just before reconstruction while minimizing ringing artifacts that may arise from the reconstruction process. By leveraging the U-Net model, the research aims to enhance segmentation consistency and preserve complex anatomical details in medical imaging applications. The main contribution and motivation of the current study can be summarized as follows. SPECT myocardial perfusion imaging (MPI) presents significant challenges, including low resolution, noise, and anatomical variability, making segmentation more complex compared to other imaging modalities like CT or X-ray. To address these challenges, a U-Net model was explicitly trained and adapted for SPECT MPI.

Additionally, the study introduces a unique dataset of 4560 images and masks (derived from 1043 MPI scans), manually labeled by nuclear medicine experts, providing a reliable foundation for model training and evaluation. This study utilizes collaborative error analysis of the results, which demonstrate the robustness and efficiency of the proposed deep learning-based SPECT segmentation method. Furthermore, this study aims to use the U-Net model to significantly reduce manual segmentation workload and variability, offering improved consistency and efficiency for clinical applications.

## 2. Materials and Methods

This section provides an overview of the databases utilized in this study and outlines the proposed U-Net framework.

### 2.1. Dataset

#### 2.1.1. Data Collection

A total of 1100 myocardial perfusion imaging (MPI) studies conducted between 2015 and 2024 were retrospectively selected, involving patients aged 17 to 88 years. A sample size of 1100 cases ensures adequate power to detect significant differences or relationships, ensure robust U-NET training, prevent overfitting, and effectively capture variability in medical image segmentation tasks. Approximately 57 cases were excluded due to inadequate image quality, characterized by a poor target-to-non-target ratio. All images (64 × 64 matrix size) were obtained in the supine position, utilizing an 8-frame electrocardiogram (ECG)-gated, 2-day rest/stress protocol. The imaging was performed using the GE SPECT/CT Optima NM/CT 640 gamma camera equipment, manufactured by GE Healthcare (Waukesha, WI, USA). Raw data for analysis were retrieved from the Picture Archiving and Communication System (PACS) at the Nuclear Medicine Department of Al-Jahra New Hospital, (Jahra, Kuwait). The radiopharmaceutical used was technetium Tc-99m-myoview (also known as ^99m^Tc-tetrofosmin), with a standard dose of 20 mCi, administered according to Clark’s formula. The ordered subset expectation maximization (OSEM) algorithm, with 2 iterations and 10 subsets, was used to reconstruct the SPECT images. Since this is a machine learning study, normality tests are not typically required as ML models do not assume specific data distributions. The data was then categorized into training set (*n* = 100 images) and a validation set (*n* = 900 images). 

#### 2.1.2. Labeling

**Figure 2:**
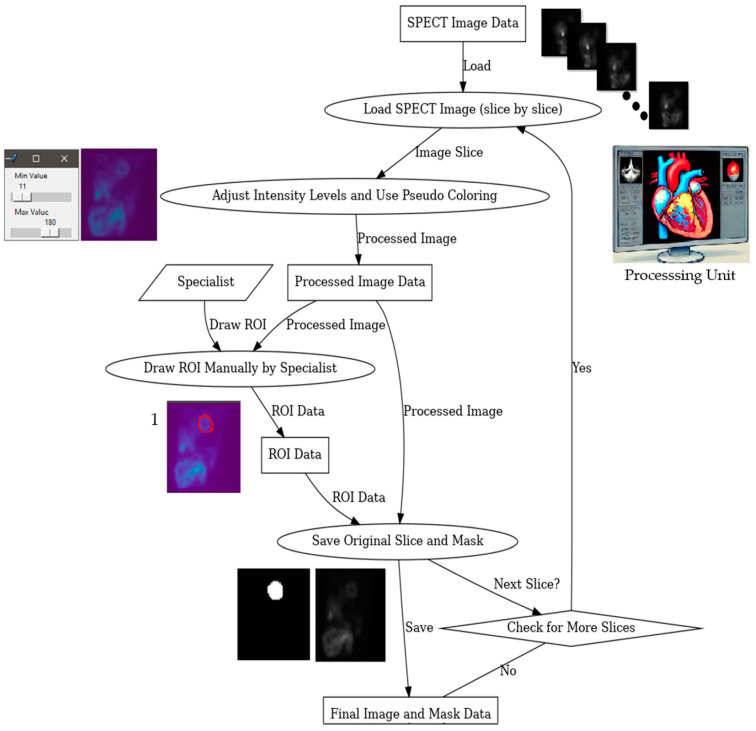
The full labeling process. The red circle in image 1 indicates the ground truth region of interest.

To label the data (compute the ground truth masks), the collected SPECT images were manually labeled by three experts based on a semiautomated Python code. The semiautomated code represents a friendly simple graphical user interface GUI, which is prepared using the “Tkinter” library. In this simple GUI, the specialist labeler is requested to load one 3D SPECT scan and then the GUI displays the slices sequentially, allowing the specialist to manually draw a free-hand polygon around the ROI and then continue to the next slice. After finishing each 3D SPECT file, the original images with their corresponding created masks will be shown in the same directory of the working file. Figure 2 shows an illustration of the entire labeling process.

#### 2.1.3. SPECT Dataset

After data acquisition and labeling, the dataset comprises a total of 4560 images and corresponding masks. These images were meticulously split into three distinct sets to ensure comprehensive training and evaluation of the model: the training set contains 3488 images and corresponding masks, the validation set includes 616 images and masks, and the test set consists of 456 images and masks. The primary challenge of this dataset lies in the unclear regions of interest (ROIs), which are located at varying coordinates and exhibit low contrast. High contrast in other parts of the images could potentially interfere with the learning process. However, deep learning models, particularly the U-Net architecture, are well suited to learn from such challenging data.

### 2.2. Data Preprocessing and Augmentation

Given the inherent challenges posed by the dataset—such as the low contrast of the regions of interest (ROIs) and their varying coordinates—additional preprocessing steps were crucial. These steps included normalizing the pixel values of the images and ensuring the images were resized to a uniform dimension of 64 × 64 pixels. This resizing facilitated efficient processing by the U-Net model while preserving essential details necessary for accurate segmentation. The grayscale images simplified the data and reduced computational complexity without compromising the critical information required for the segmentation tasks.

The augmentation methods involved horizontal and vertical flips, which randomly flipped the images along either axis, thereby doubling the training data. This approach helps the model become resistant to orientation changes, enhancing its ability to identify ROIs regardless of their position in the image. Grid distortion was applied with a probability of 0.2, introducing random perturbations to the images. This technique simulated real-world distortions and made the model more resilient to minor deformations and variations in the input data.

**Figure 3:**
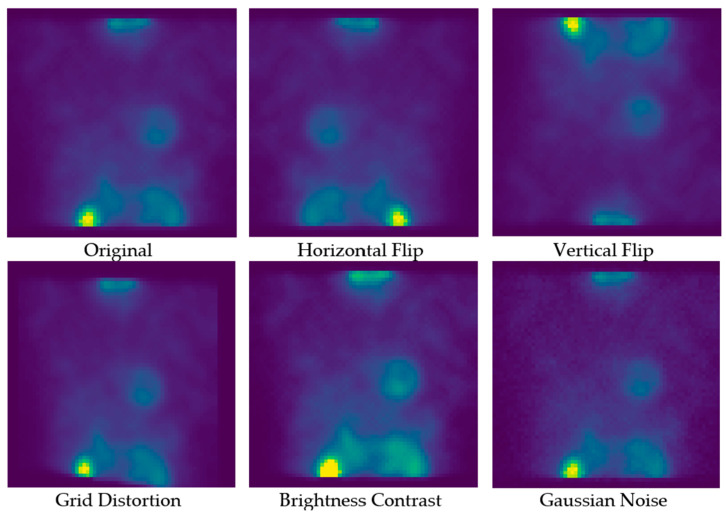
The proposed data augmentation operations were applied to a training sample (images are colored using a pseudo color to clarify the differences between these various copies).

Furthermore, random changes in illumination and contrast, within a range of 0 to 0.5, were applied to the images. This augmentation increased the model’s resilience to varying grayscale conditions and contrast levels, which is essential for maintaining consistent performance under different imaging conditions. Additionally, Gaussian noise with a mean of 0 and a standard deviation of 0.5 was added to the images, further improving the model’s ability to handle noisy inputs. This augmentation ensured that the model could accurately segment ROIs even in the presence of image noise, a common challenge in real-world medical imaging scenarios. The augmentation process ensured that the U-Net model remained effective and reliable across different subsets of the data, ultimately leading to improved segmentation performance and generalization. Figure 3 shows an example of applying these data augmentation operations to the training data.

By integrating these preprocessing and augmentation steps, the data processing pipeline effectively prepared the dataset for training, leading to the development of a robust and accurate segmentation mode.

### 2.3. Proposed U-Net Model

The proposed model for this study is a U-Net architecture renowned for its effectiveness in semantic segmentation tasks, particularly in medical imaging. The U-Net model is designed to perform pixel-wise segmentation, which is crucial for accurately identifying regions of interest (ROIs) in medical images. The U-Net model has a symmetric architecture, comprising a contracting path to capture contextual information and a corresponding expanding path for accurate localization. The contracting path follows the structure of a standard convolutional network, involving repeated convolutions, followed by a rectified linear unit (ReLU) and max-pooling operations. This path is responsible for extracting high-level features and reducing the spatial dimensions of the input image, thereby capturing key features essential for segmentation.

On the other hand, the expanding path is designed to enable precise localization through transposed convolutions, which restore the spatial dimensions reduced during the contraction phase. This path also incorporates concatenations with the corresponding feature maps from the contracting path, providing fine-grained information that helps in achieving accurate segmentation. The U-Net model employed in this study consists of several layers, each performing specific operations such as double convolutions, max pooling, and transposed convolutions. The input to the model is an image sensor with dimensions tailored to the dataset (batch_size = 128; num_channels = 3; height = 64; width = 64). The network’s depth allows it to capture a wide range of features at different scales, improving its capability to accurately segment complex structures within the images.

In addition to its architectural design, the model incorporates several training strategies to enhance performance. These include using data augmentation techniques to artificially expand the training dataset, applying a cross-entropy loss function to measure segmentation accuracy, and adopting the AdamW optimizer to update the model weights efficiently. The training process is further supported by mechanisms such as early stopping to prevent overfitting and saving the best model based on validation performance. This aims to achieve a robust and precise segmentation model capable of accurately delineating ROIs even in challenging imaging conditions.

### 2.4. Evaluation Metrics

**Figure 4:**
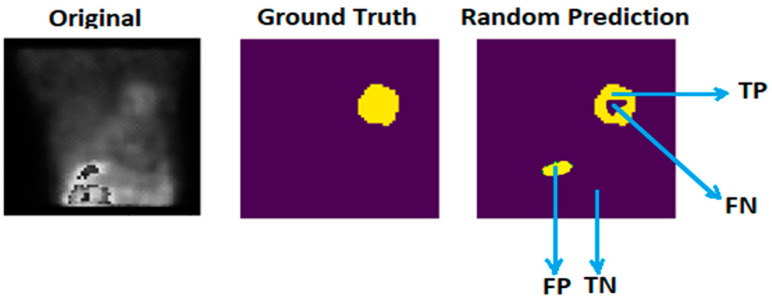
Original is the raw data image, ground truth is the mask provided by experts, and random prediction is true positives (TPs), true negatives (TNs), false positives (FPs), and false negatives (FNs) in a random supposed segmentation result of a sample of the utilized dataset. TPs indicate correctly predicted foreground pixels, while TNs indicate correctly predicted background pixels. FPs are background pixels incorrectly predicted as foreground, and FNs are foreground pixels incorrectly predicted as background.

The effectiveness of the proposed U-Net model is demonstrated by its high performance across various evaluation metrics, including intersection over union (IoU), Dice score, pixel accuracy, precision, and recall, underscoring its suitability for medical image segmentation tasks [17,18,19,20,21]. All metrics are derived from the four main calculations of the segmentation result (see Figure 4).

Precision indicates the frequency with which predictions for the positive class are accurate in the segmentation output, whereas recall reflects the model’s ability to identify all positive pixels in the segmentation result. The formulas for precision and recall are provided in Equations (1) and (2).

(1) $$Recall=\frac{TP}{TP+FN}$$

(2) $$Precision=\frac{TP}{TP+FP}$$

**Figure 5:**
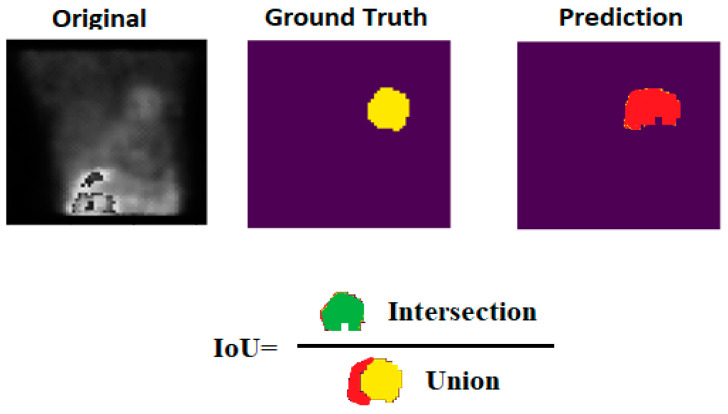
IoU example on a sample of the dataset and its corresponding prediction.

IoU calculates the overlap between the predicted segmentation mask and actual ground truth mask (see Figure 5), with scores ranging from 0 to 1, and is given as the formula below shows:

(3) $$IoU=\frac{TP}{TP+FP+FN}$$

Dice is a measure of the similarity between the ground truth and the predicted segmentation. The Dice score has a range between 0 and 1 and is given as the following formula:

(4) $$Dice=\frac{2*TP}{TP+FP+TP+FN}$$

Pixel accuracy indicates how many pixels in the predicted segmentation map match the ground truth labels and are expressed as follows:

(5) $$Pixel\;Accuracy=\frac{TP+TN}{TP+FP+TP+FN}$$

## 3. Experiments

The training process involved feeding the training pairs (images and masks) into the U-Net model. The model was trained to establish the relationship between the input images and their corresponding masks. The training settings included 100 epochs, an initial learning rate of 1 × 10^−4^, a weight decay of 1 × 10^−6^ to regulate weight adjustments during training, and a cross-entropy loss function optimized with the AdamW optimizer. To improve the model’s robustness, data augmentation techniques such as horizontal and vertical flips, grid distortion, random variations in illumination and contrast, and the addition of Gaussian noise were applied.

Table 1 summarizes the training options utilized in our experiments.

### 3.1. Results

**Figure 6:**
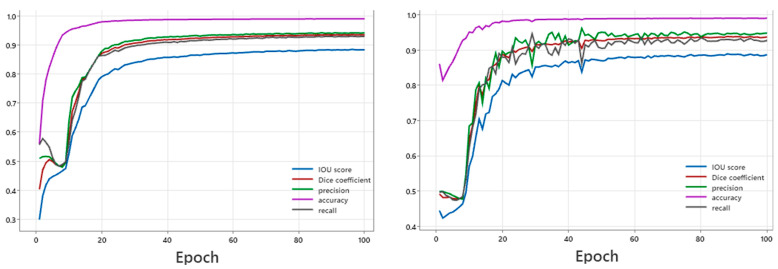
Model performance showing multiple iterations of epochs for the training set (**left** graph). Model performance increases with multiple iterations of epochs of the validation set (**right** graph).

The training and validation curves showed decreased loss and increased pixel accuracy, IoU, Dice score, precision, and recall over the epochs, indicating effective training without overfitting. After training, the model was tested on the test set to evaluate its generalization performance. The results demonstrated high segmentation accuracy, with the test set achieving an mIoU of 0.896, a Dice score of 0.94, and a pixel accuracy of 0.989 (see Figure 6 and Table 2 for more details). These metrics were consistent with the performance on the training and validation sets, highlighting the model’s robustness and effectiveness.

**Figure 7:**
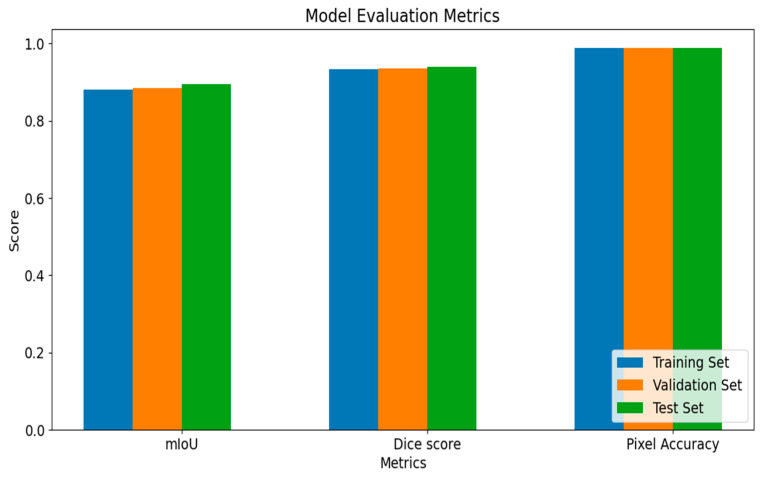
Model evaluation metrics for all sets.

**Figure 8:**
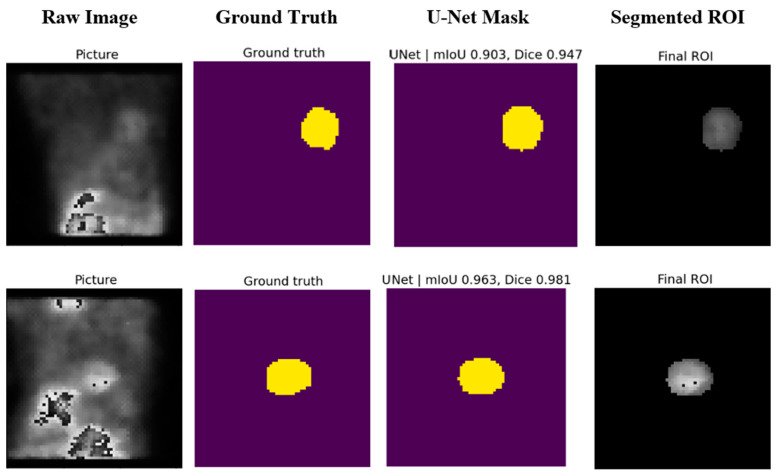
Actual example of some random SPECT projection segmentation results via U-Net.

Figure 7 shows a bar plot of the metrics for the training, validation, and test sets for a more precise comparison. The average mIoU values for training, validation, and test sets are 0.881, 0.885, and 0.895, respectively. For the Dice score, the model achieved 0.933, 0.936, and 0.94, respectively, while for the pixel accuracy score, we obtained 0.989 for all sets (see Figure 8).

Visual tests were conducted on the test samples to further assess the model’s capability. The predicted masks were compared with the ground truth masks, and the segmented ROIs were examined. The visual results confirmed the model’s ability to accurately predict the ROIs even in challenging cases with low contrast and unclear boundaries. 

### 3.2. Confusion Matrix

**Figure 9:**
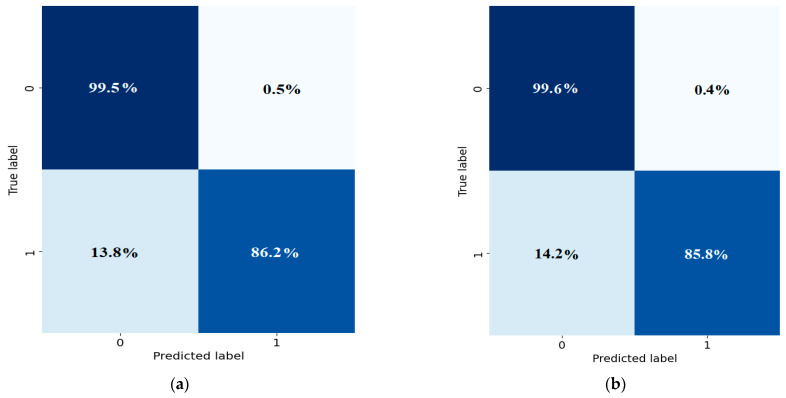
Confusion matrices for model evaluation: true vs. predicted labels. (**a**) For training data; (**b**) for test data.

The confusion matrix displays the outcomes of predictions, where false positives (FPs) occur when the model incorrectly predicts a positive class for a true negative, and false negatives (FNs) occur when the model misclassifies a true positive as negative. To evaluate the classification performance of the U-Net model in detail, we present its corresponding confusion matrix. This 2 × 2 matrix shows the distribution of true labels versus predicted labels for myocardium segmentation (Figure 9). It offers an in-depth view of the model’s classification performance, enabling a thorough assessment of how accurately the model performs the segmentation. Figure 9 shows that the trained U-Net model is capable of predicting the ROI and background pixels with 99.5% and 86.2% and 99.6% and 85.8% true positive rates for both training and test datasets, respectively.

### 3.3. Receiver Operator Characteristic

**Figure 10:**
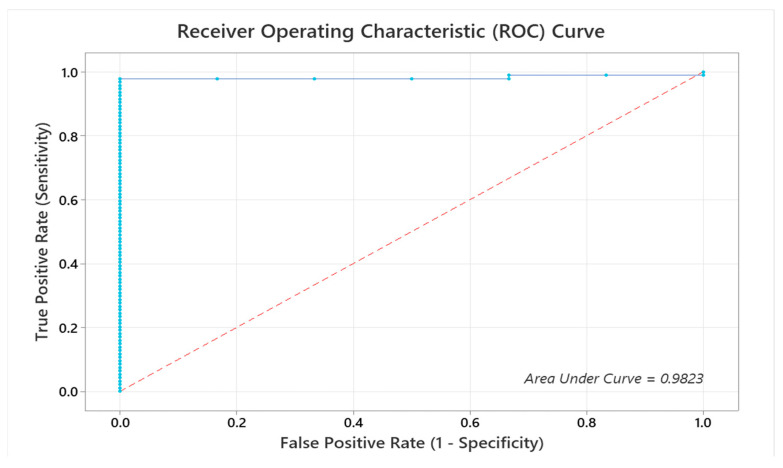
ROC curves illustrating model performance: individual class ROC curves.

To thoroughly evaluate the discriminative ability of our models across all segmentation classes, we present Receiver Operating Characteristic (ROC) curves. The ROC curve combines the true positive and false positive rates across these classes, offering a complete assessment of the model’s overall performance. The binary logistic regression analysis examined the relationship between recoded validation recall values and validation true positives (val_TP). The analysis used 100 rows of data (100 epochs), with 94 events. The coefficient for val_TP was 0.0114, with a standard error of 0.0031, a Z-value of 3.70, and a *p*-value > 0.0001, indicating strong statistical significance. The odds ratio for val_TP was 1.0115, with a 95% confidence interval (CI: 1.005–1.017). The model explained 43% of the deviance, with an adjusted R-squared of 40%. The area under the ROC curve was 0.98, demonstrating excellent model discrimination (see Figure 10).

### 3.4. Comparative Analysis

**Figure 11:**
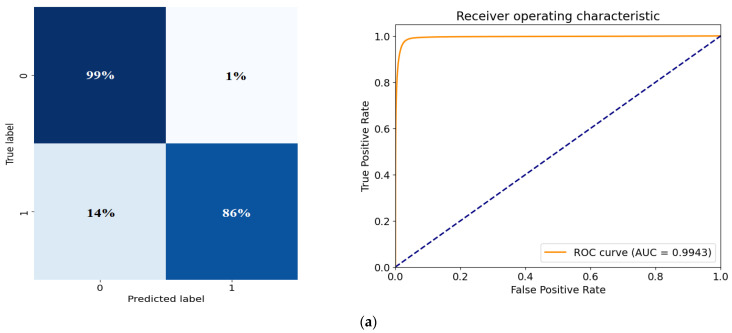

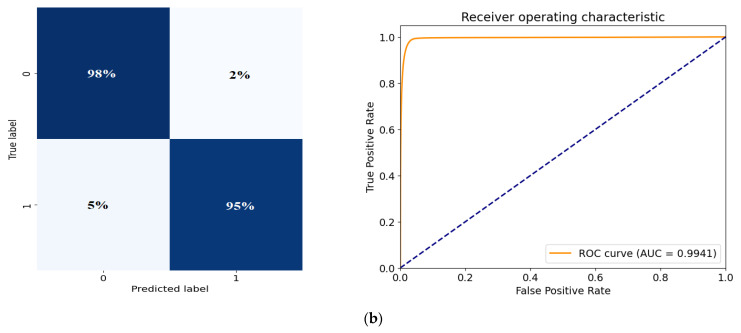
Confusion matrix and ROC plot for the extra scenarios: (**a**) k-fold split scenario, (**b**) k-fold with Dice loss scenario.

In this part, two scenarios were carried out. In the first one, the data split concept was changed for a more deep analysis study. The well-known k-fold split concept [21] was utilized. In this scenario, we first select 10% of the dataset as a test set, then we utilized the 5-fold (K = 5) splitting concept in which the dataset is split into 5 separate folders (1 of them is used for validation, while the remaining 4 folders are considered as a training set). As a result, the training, validation, and test sets included 3284 images, 820 images, and 456 images, respectively. In the second scenario, the cross-entropy loss was replaced by the Dice loss, and the k-fold split was also maintained. Figure 11 includes the confusion matrix and ROC plot of both k-fold splitting and k-fold with Dice loss-based scenarios.

**Figure 12:**
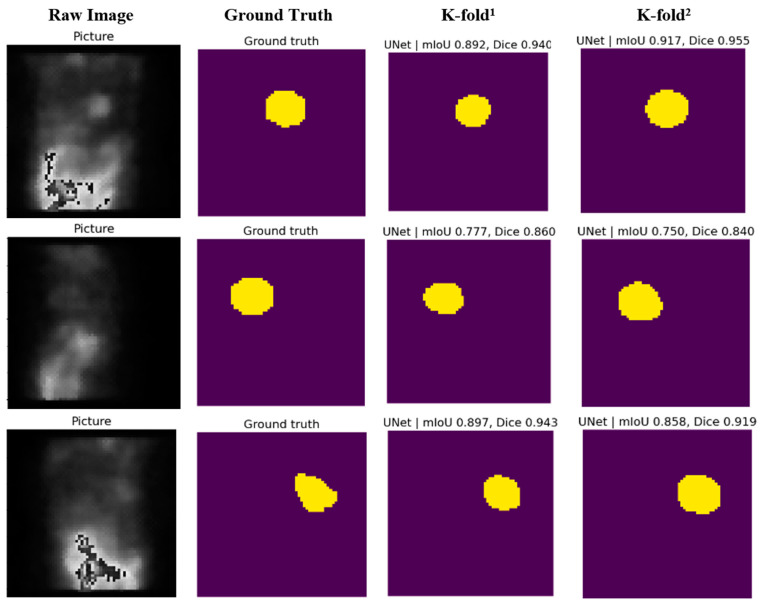
Actual example of SPECT frame segmentation results via U-Net (k-fold and Dice loss experiments). **K-fold^1^** represents k-fold cross-entropy loss mask, and **K-fold^2^** represents Dice loss mask.

**Figure 13:**
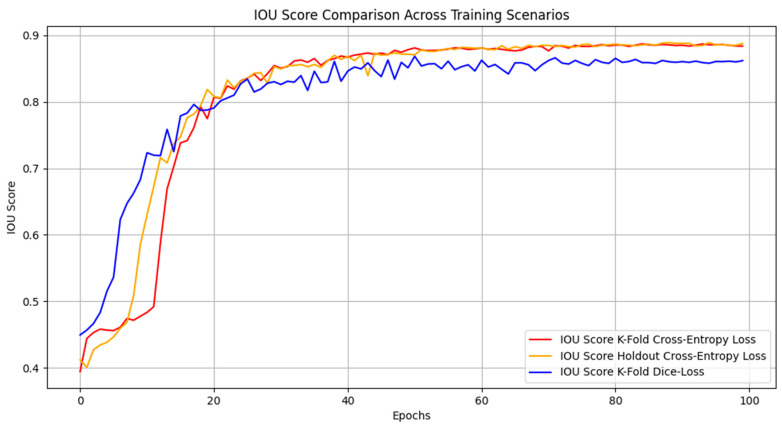

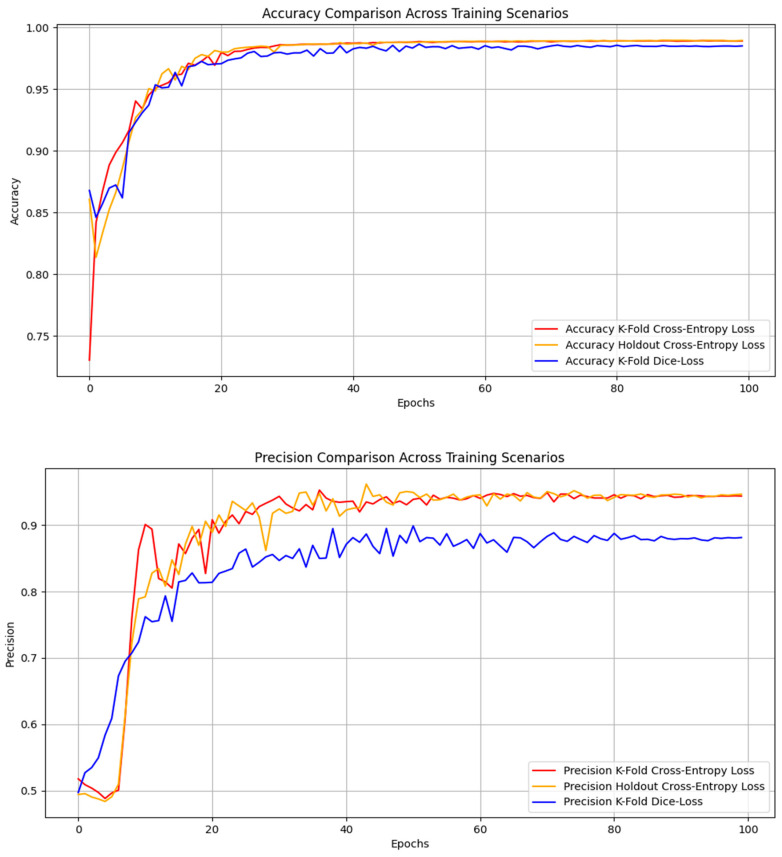

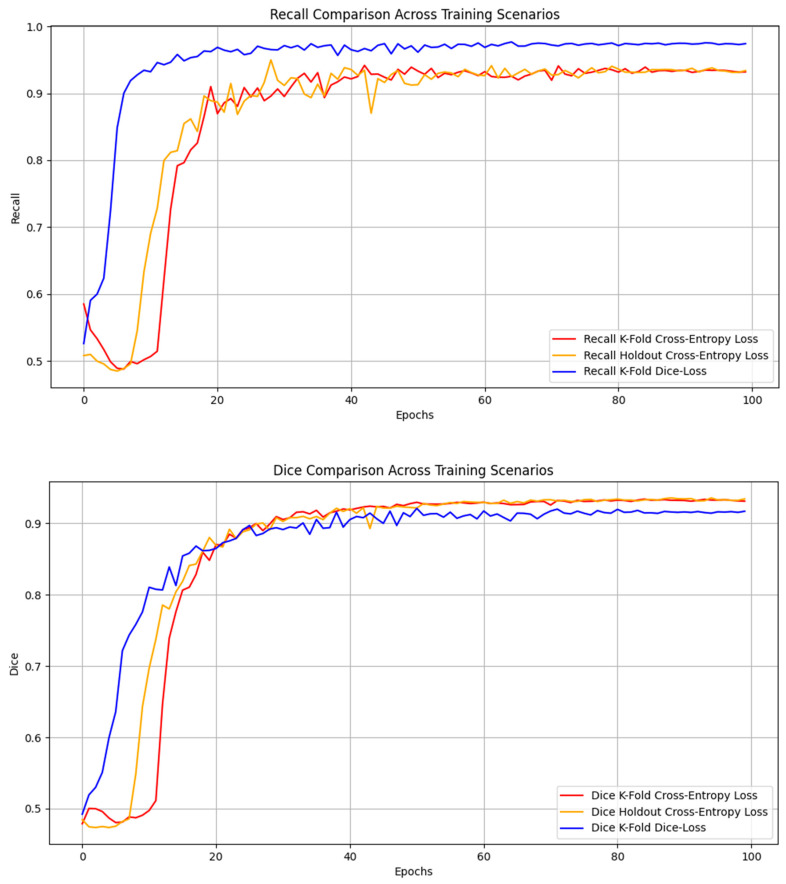
Model performance using different loss functions and split concepts (entropy loss, Dice loss, and k-fold split).

The confusion matrix and ROC plot of the k-fold split dataset has a slightly lower performance compared to the original holdout train–validation–test split scenario. The mIoU, Dice, and accuracy scores are 0.8767, 0.9273, and 0.9872, respectively. The AUC score of the k-fold method is 0.99, while the AUC of the holdout concept is 0.98, indicating that the k-fold method has a slightly better balance between TPs and FPs. Figure 12 includes visual segmentation results of the trained U-Net model using the k-fold split scenario. Moreover, for the k-fold with a Dice loss training scenario, the confusion matrix looks more balanced (both ROI and background classes have similar metrics), which is better than the original and the k-fold with cross-entropy loss scenarios. However, the k-fold with cross-entropy scenario outperforms the k-fold with Dice loss scenario in terms of TPs and FNs of the ROI class leading to better metrics of mIoU, Dice, and accuracy scores (0.845, 0.9063, and 0.981, respectively). For a more detailed comparison, Figure 13 shows a comprehensive comparison between the three proposed training scenarios under all performance metrics.

Since the Dice loss improved the TPs and FNs of the ROI class, the recall curve of the Dice loss training scenario is better than the other ones (with cross-entropy loss). This is normal since the recall formula is TP/(TP + FN) and since the TP is increased and the FN is decreased, the overall recall is enhanced. However, since the FP is increased, the precision curve (TP/(TP + FP)) of the Dice loss scenario is worse than the corresponding precision curves of the cross-entropy loss (see Figure 13). The rest of the metrics are almost the same in all scenarios which proves the same conclusion that all training scenarios are robust and have a close segmentation performance.

### 3.5. Collaborative Error Analysis and Dataset Refinement

**Figure 14:**
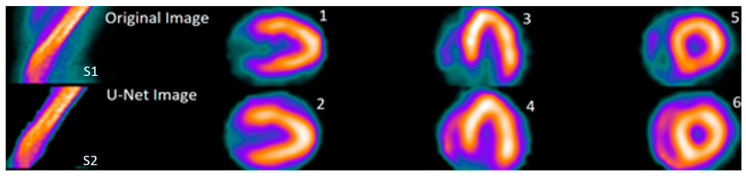
An image representing corresponding sinograms (S1 and S2) and various axes of original and U-Net images. Images are vertical long axis (1 and 2, for original and U-Net images, respectively), horizontal long axis (3 and 4, for original and U-Net images, respectively), and short axis (5 and 6, for original and U-Net images, respectively). Original images were processed using Myovation^®^ segmentation while U-Net images were segmented using U-Net code.

Comparing the reconstruction results of the U-Net model with those of the clinical software Myovation^®^ package, 2018 (GE Healthcare, Chicago, IL, USA), the results demonstrate similar axes and closely corresponding values (*p*-value = 0.1) (see Figure 14 and Table 3). Although we did not perform a systematic qualitative analysis, the consultant physician reported a good quality in spite of the slight over-smoothing.

In our evaluation of the proposed image segmentation model, we analyzed 100 cases and identified 3 instances of misclassification. It was found that the misclassification occurred due to noise and inaccurate labeling.

### 3.6. Clinical Parameters and Qualitative Images and Integration with Clinical Work

**Figure 15:**
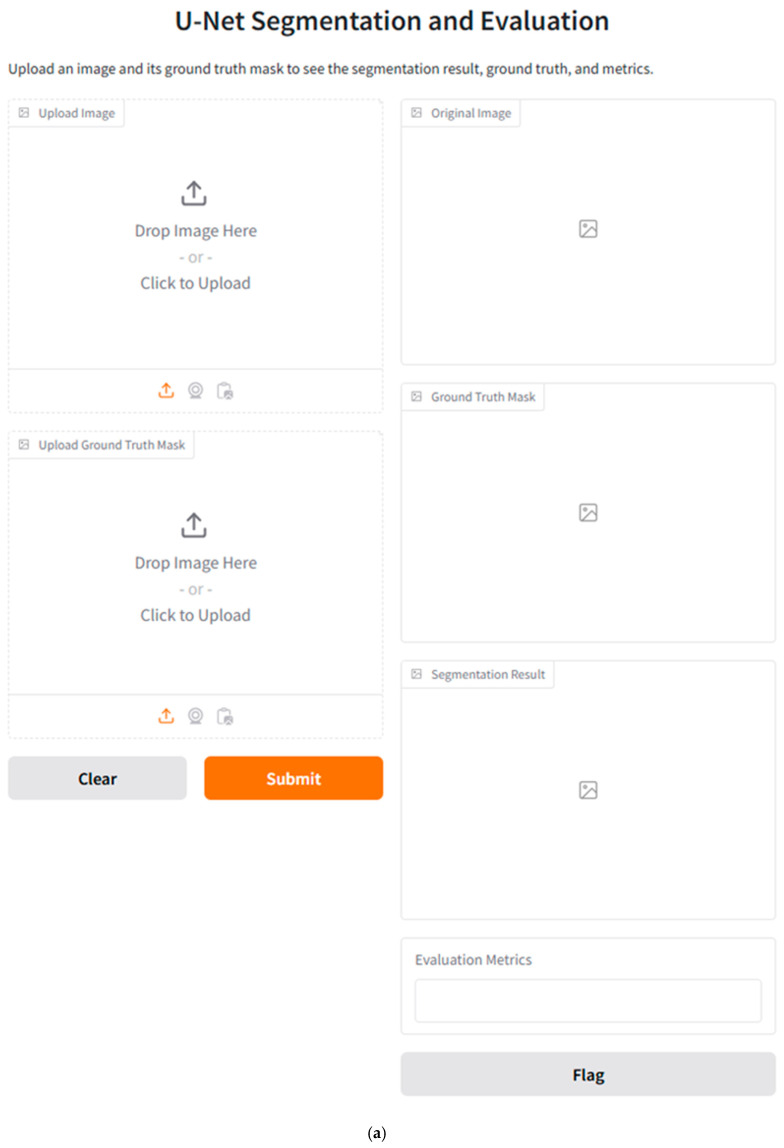

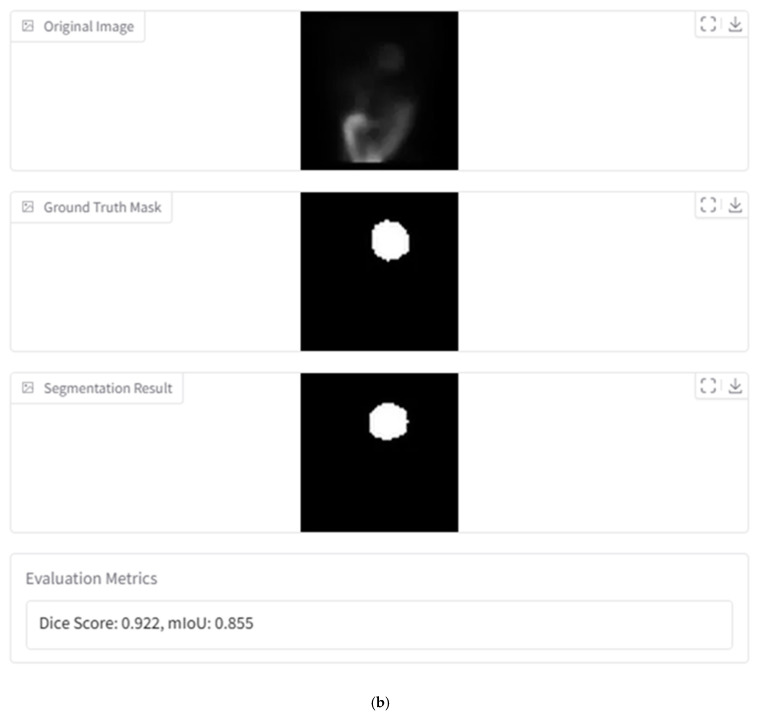

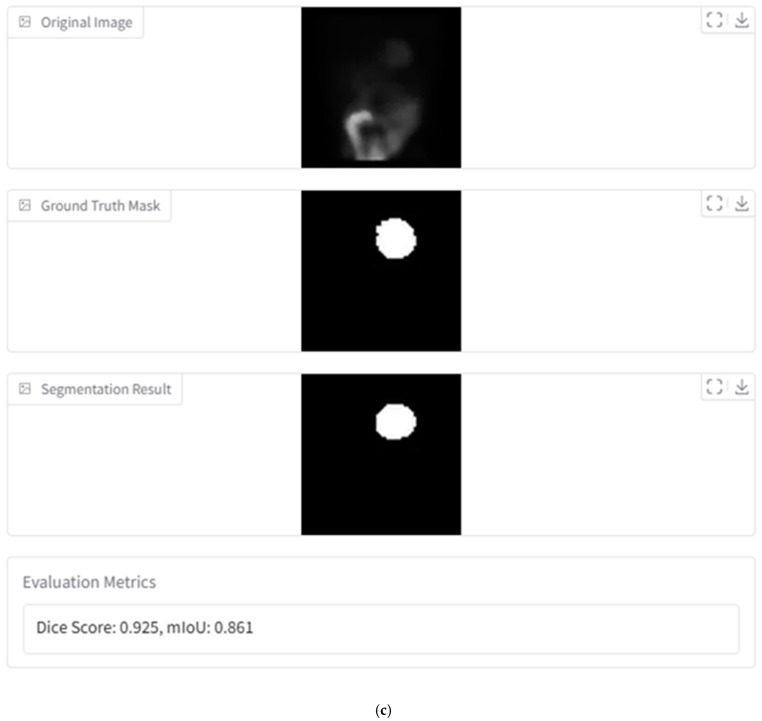
The designed GUI: (**a**) the main design, (**b**) test example 1, and (**c**) test example 2.

Figure 15 presents the main design of this GUI and two test examples applied to two different test samples. The figure is designed to ease the process of image labeling and further segmentation.

### 3.7. Comparison with Other Segmentation Frameworks

In this section, the U-Net model’s performance is compared to other advanced semantic segmentation models using our collected dataset. Table 4 shows the performance metrics of U-Net and three other semantic segmentation models (Deeplab_resnet50, DeepLabV3+ and SegNet), which are famous for their high performance. However, the proposed U-Net model outperforms the other two models in terms of all metrics (accuracy, precision, recall, F1 score, and IoU score).

## 4. Discussion

### 4.1. Results and Discussion

Unlike much of the ML research that focuses on disease classification and uses conventional ways to segment the myocardium in SPECT MPI [12,22,23,24,25,26,27,28], our paper has introduced an innovative way to segment the myocardium right before the reconstruction. We conducted a thorough analysis of U-Net models; it is extremely feasible to include such a model for automatic or semiautomatic segmentation in SPECT cardiac imaging before running a classification model. This will help process most of the mega data and assist in extracting as many radiomic features from images as possible for clinical correlation and classification. Our methodology was rigorously evaluated on the Jahra Hospital dataset, encompassing > 1000 SPECT scans from various patients with various cardiac conditions, aligning with established categories in the literature [12,22,23]. Using a local dataset for ML segmentation ensures models are tailored to regional patient characteristics, imaging protocols, and clinical workflows, improving accuracy, relevance, and trustworthiness. Furthermore, accurate ML segmentation avoids the variability resulting from manual or semiautomated segmentation created by multiple experts [29,30]. The reconstructed images from U-Net represent a high target-to-non-target ratio image that could provide a more straightforward qualitative assessment by physicians.

The significance of our U-Net model lies in its pursuit of heightened accuracy and performance in SPECT image segmentation, easing and standardizing the process of cardiac image segmentation. Notably, the U-Net model surpassed manual segmentation, demonstrating good image quality while saving time [14]. Most researchers do not focus on segmentation but rather try to build up a prediction or diagnosis model without focusing on the segmentation [25]. In fact, Kusumoto reported that despite significant advancements in deep learning, limitations such as the loss of information from using 2D input data and insufficient training datasets due to limited cases of CAD lesions hinder diagnostic accuracy. This limitation can be overcome by using most of the available images by utilizing ML segmentation techniques before feeding images to the diagnostic model. Crucial to our success were data preprocessing and augmentation methods, including resizing, normalization, random cropping, flipping, rotation, transformation, brightness adjustment, and scaling. Our study on the U-Net model for SPECT MPI image segmentation has shown promising results that align with and, in some cases, exceed those found in similar research [12,14]. Our study on the U-Net model for SPECT MPI image segmentation achieved a Dice score of 94% and pixel accuracy of 98.9%, surpassing the 89% precision reported by Szűcs et al. [23] for their 3D U-Net-based self-supervised learning approach. Both studies demonstrated robust performance, with high AUC values of 0.98, underscoring the effectiveness of the 3D U-Net architecture in medical imaging. Our model’s superior metrics may be attributed to comprehensive data augmentation and optimized training parameters. These findings confirm that advanced U-Net architectures and tailored training strategies significantly enhance SPECT MPI segmentation, offering reliable tools for clinical applications.

In fact, our result constitutes many difficulties as compared to studies that utilized reconstructed images (e.g., Szűcs et al. [23]). Our study performed the segmentation on the raw data projection of SPECT MPI images instead of reconstructed axes. This added many difficulties concerning afterward reconstruction, which led to ringing. This occurs due to the abrupt transition between the high-activity region (organ of interest) and the black mask (background). When the reconstruction algorithm encounters these sharp edges, it can create high-frequency artifacts or ringing effects. This happens because the algorithm is trying to fit the sudden changes in intensity, leading to artificial high uptake around the margins of the mask. To minimize this artifact, we applied a smoothing function to the mask edges for a gradual transition between the organ and background, reducing sharp intensity changes [8,9,24]. A soft mask was also investigated with gradually decreasing intensity values toward the edges to avoid abrupt transitions. Additionally, post-processing filters like Gaussian smoothing or median filters were implemented in the reconstructed images to reduce high-frequency artifacts. All these factors were evaluated while maintaining high clinical image quality.

Similar to many related studies [14,22], our research has significant implications for SPECT MPI and healthcare AI. The proposed U-Net model shows promise for improving SPECT MPI image segmentation, potentially improving the analysis of large datasets, and advancing the field of radionics in nuclear medicine [14,22]. The increased segmentation accuracy can positively impact patient outcomes by allowing for better and standardized clinical parameter calculations. Additionally, analyzing mega data in this way will aid in understanding artifacts and false positive sources of error during SPECT MPI.

The U-Net model’s reconstruction results were closely aligned with those of the clinical software (Myovation^®^, GE Healthcare), with similar axes and corresponding values (*p*-value = 0.1). Despite a slight over-smoothing, the consultant physician reported good quality. In a significant development for our collaborative project, we thoroughly examined cases that were initially misclassified by our proposed model. Out of the 100 cases initially evaluated, our analysis revealed 3 instances of misclassification. This initial phase of evaluation provided crucial insights into the performance and robustness of our model and allowed us to investigate the reason thoroughly. This investigation encompassed examining both the ground truth labels and predicted labels, as well as closely inspecting the frames within the mislabeled cases. Several noteworthy observations emerged from this review, underscoring the exceptional capabilities of our model. Firstly, we identified discrepancies in ground truth labeling, where entire volumes were inaccurately labeled due to Poisson noise. For instance, in the case of one scan, the masking of some frames was erroneously skipped. Subsequent rectifications were made to resolve these errors throughout the dataset. Rectifications included denoising and contrast enhancement before segmentation. Secondly, instances were found where our model accurately detected the presence of activity outside the myocardium region, while the model erroneously included these regions as myocardium.

This investigation was thoroughly performed by experienced image analysts and experienced nuclear medicine technologists and physicians. This expertise played a pivotal role in identifying and rectifying such discrepancies. Lastly, it was acknowledged that some scans in the dataset posed significant challenges due to excessive noise or the presence of conditions such as poor target-to-non-target ratios. To avoid concerning misclassification, problematic cases were excluded from further analysis. As a result of these corrections, our model’s accuracy rate substantially improved to >94%, with only a few remaining misclassified cases. These developments significantly enhance the robustness and reliability of our proposed model and underscore the importance of close collaboration between medical experts and machine learning practitioners in the realm of healthcare AI research [31].

Our results demonstrate that the U-Net model outperforms DeepLabV3+ and SegNet in semantic segmentation on our dataset, achieving the highest scores across all metrics. These findings are consistent with the results reported by Hadinata et al. [32]. U-Net’s architecture, with its combination of down-sampling, up-sampling, and skip connections, enables precise segmentation and boundary delineation, making it particularly effective for this application [22]. These findings highlight U-Net as the most reliable and robust model for our segmentation task.

### 4.2. Integration into Clinical Work

The proposed segmentation framework supported by the U-Net model can be integrated into an image processing pipeline using the SPECT MPI images. Since the trained U-Net model can automatically segment SPECT images, it can provide clinicians with delineated myocardial regions for later analysis steps. Moreover, the high speed of the segmentation operation allows the trained U-Net model to be integrated into real-time consultations or pre-reporting processes. The original SPECT images can be compared to the segmentation results of the proposed U-Net model side by side and serve as a decision-support tool to help physicians make their medical decisions more efficiently.

To improve the utility of the trained U-Net model, clinicians need to concentrate training on understanding the AI-generated results (i.e., understanding the IoU and Dice coefficients since these metrics reflect the confidence scores of the model’s results). However, clinicians should be aware of the cases in which the segmentation tool can produce inaccurate or incomplete results (i.e., high noise rate). Furthermore, the designed tool is not a replacement for the physician or clinician’s opinion. For previous reasons, we designed and implemented a graphical user interface GUI using the well-known “Gradio” Python framework to facilitate the interaction with our proposed model.

### 4.3. Shortcomings of Previous Relevant Studies

While some studies include a range of patient categories [25,26,27,28], the sample’s representativeness concerning the broader population needs to be fully addressed, which may affect the generalizability of the findings. Unlike Berkaya et al. [28], our study’s generalizability, by using a large, diverse dataset encompassing various patient categories, including healthy and unhealthy individuals across different age groups, as well as cases with noisy data and suboptimal target-to-non-target ratios, ensures the model’s robustness and applicability across real-world scenarios. The model’s performance in many studies is not validated on an independent external dataset, raising concerns about its applicability to different populations or clinical settings. Unlike previous studies focusing solely on predictive modeling [27,28,29], our study incorporates a user-friendly GUI, enabling physicians and non-expert researchers to efficiently utilize the tool for segmentation, ground truth determination, and model evaluation for research applications in clinics.

## 5. Limitations

We selected a sample size of 1100 cases to ensure diverse representation, including healthy and unhealthy individuals across all age groups, as well as cases with noisy data and suboptimal target-to-non-target ratios. This large and varied dataset helps minimize selection bias by capturing the natural variability in SPECT imaging, reducing the risk of overfitting, and ensuring the model’s generalizability to broader populations. However, the study is limited by its retrospective design, which may introduce inherent biases related to data collection or selection. Future studies with prospective designs could further validate the findings and address potential limitations of retrospective data. Furthermore, we emphasize the importance of clinician awareness of the model’s limitations, such as susceptibility to noisy or low-quality data, and the utility of confidence metrics (e.g., IoU and Dice coefficients) in interpreting results.

## 6. Conclusions

In conclusion, our study provides an in-depth exploration of AI-driven advancements in image segmentation using SPECT MPI images. Through a detailed analysis of the U-Net model, its associated hyperparameters, data augmentation techniques, and error correction methods, our research highlights the potential of AI as a valuable asset in nuclear cardiology. Our results emphasize the importance of high-quality data and expert collaboration in creating effective ML solutions for healthcare, particularly in nuclear medicine image analysis. As AI continues to evolve in healthcare, focusing on interpretability, generalization, and ethical concerns, our work contributes to the ongoing efforts to enhance diagnostic accuracy and patient care. The collected SPECT dataset supported by an expert labeling process constitutes a very essential field for future studies. Moreover, the comparison of U-Net’s SPECT segmentation performance to that of experts achieved almost similar performance (*p* = 0.1), which proves its clinical feasibility and validity for real-world integration. Moreover, comparing the proposed methodology with other recent semantic segmentation frameworks proved its robustness and transcendence.

## Figures and Tables

**Table 1:** Training parameter details.

Parameter	Description
Training epochs	100
Initial learning rate	1 × 10^−4^
Weight decay (control weight changes)	1 × 10^−6^
Loss function	Cross-entropy
Optimizer	AdamW (Adam with weight decay)
Saving best model	Yes
Early stopping of training	Yes
Image and mask dimensions	64 × 64
Batch size (number of images per training step)	128

**Table 2:** Descriptive statistics for evaluation metrics of the U-Net model for the training set and validation set, including mean and standard deviation scores, in addition to the minimum–maximum range.

Training Set					
	IOU Score	F1	Precision	Accuracy	Recall
mean	0.81	0.9	0.9	0.96	0.86
std	0.14	0.13	0.13	0.1	0.12
range	0.30–0.90	0.40–0.93	0.50–0.94	0.60–0.98	0.60–0.92
**Validation Set**					
mean	0.81	0.87	0.88	0.97	0.86
std	0.13	0.13	0.13	0.03	0.13
range	0.445–0.88	0.5–0.93	0.5–0.94	0.86–0.98	0.5–0.93

**Table 3:** Corresponding total and mean gray values of vertical long axis (1 and 2, for original and U-Net images, respectively), horizontal long axis (3 and 4, for original and U-Net images, respectively), and short axis (5 and 6, for original and U-Net images, respectively). Original images were processed using Myovation^®^ segmentation while U-Net images were segmented using U-Net code.

Region	Total Gray Value	Mean Gray Value	LVEF
1	8938	110	U-Net LVEF = 64% Original software LVEF = 63%
2	8472	131	
3	6934	128	
4	7795	136	
5	5740	128	
6	5882	147	

**Table 4:** Comparing U-Net performance with other semantic segmentation models.

	IOU Score	F1	Precision	Accuracy	Recall
U-Net	0.81	0.9	0.9	0.96	0.86
Deeplab_resnet50	0.5910	0.7429	0.7429	0.7432	0.7429
Segformer	0.7136	0.8329	0.8329	0.8112	0.8329
Deeplabv3	0.7795	0.8761	0.8761	0.8751	0.8761

## Data Availability

The data presented in this study are openly available at https://github.com/ahmaxiom/U-Net-Deep-Learning-Model-in-SPECT-Myocardial-Perfusion-Image-Segmentation (accessed on 10 December 2024).
